# Making the Most of *In Vitro* Tests to Diagnose Food Allergy

**DOI:** 10.1016/j.jaip.2016.12.003

**Published:** 2017

**Authors:** Alexandra F. Santos, Helen A. Brough

**Affiliations:** a Division of Asthma, Allergy and Lung Biology, Department of Paediatric Allergy, King's College London, London, United Kingdom; b Children's Allergy Service, Guy's and St Thomas' Hospital, London, United Kingdom

**Keywords:** *In vitro* tests, Diagnosis, Food allergy, Specific IgE, Basophil activation test, Component-resolved diagnosis, IgG4/IgE ratio, Specific/total IgE ratio, Peptide microarray, T-cell assay, BAT, Basophil activation test, CMA, Cow's milk allergy, FA, Food allergy, kU/L, Kilounits per liter, NPV, Negative predictive value, nsLTP, Nonspecific lipid-transfer protein, OFC, Oral food challenge, PA, Peanut allergy, PFAS, Pollen-food allergy syndrome, PPV, Positive predictive value, sIgE, Specific IgE, SPT, Skin prick test

## Abstract

Various *in vitro* tests assess different aspects of the underlying immune mechanism of IgE-mediated food allergy. Some can be used for diagnostic purposes; specific IgE to allergen extracts is widely available; specific IgE to allergen components is used in most specialist centers, and the basophil activation test is becoming increasingly used clinically. IgE to allergen peptides, T-cell assays, allergen-specific/total IgE ratios, and allergen-specific IgG4/IgE ratios are currently reserved for research. Different factors can modulate the likelihood of IgE-mediated food allergy of a given allergy test result, namely, the patients' age, ethnicity, previous allergic reaction to the identified food, concomitant atopic conditions, and geographical location, and need to be taken into account when interpreting the allergy test results in the clinic. The importance of the specific food, the clinical resources available, and patient preferences are additional aspects that need to be considered when deciding whether an oral food challenge is required to reach an accurate diagnosis of IgE-mediated food allergy.

**Information for Category 1 CME Credit**

Credit can now be obtained, free for a limited time, by reading the review articles in this issue. Please note the following instructions.

**Method of Physician Participation in Learning Process:** The core material for these activities can be read in this issue of the Journal or online at the *JACI: In Practice* Web site: www.jaci-inpractice.org/. The accompanying tests may only be submitted online at www.jaci-inpractice.org/. Fax or other copies will not be accepted.

**Date of Original Release:** March 1, 2017. Credit may be obtained for these courses until February 28, 2018.

**Copyright Statement:** Copyright © 2017-2019. All rights reserved.

**Overall Purpose/Goal:** To provide excellent reviews on key aspects of allergic disease to those who research, treat, or manage allergic disease.

**Target Audience:** Physicians and researchers within the field of allergic disease.

**Accreditation/Provider Statements and Credit Designation:** The American Academy of Allergy, Asthma & Immunology (AAAAI) is accredited by the Accreditation Council for Continuing Medical Education (ACCME) to provide continuing medical education for physicians. The AAAAI designates this journal-based CME activity for 1.0 *AMA PRA Category 1 Credit*™. Physicians should claim only the credit commensurate with the extent of their participation in the activity.

**List of Design Committee Members:** Alexandra F. Santos, MD, PhD, and Helen A. Brough, MBBS, PhD

**Learning objectives**:

1. To describe the different *in vitro* tests for diagnosing food allergy and their diagnostic performance.
2. To analyze the results of allergy tests to determine the likelihood of clinical allergy.
3. To explain the factors that influence the decision to perform an oral food challenge.

**Recognition of Commercial Support:** This CME has not received external commercial support.

**Disclosure of Significant Relationships with Relevant Commercial Companies/Organizations:** A. F. Santos has received research support from the Medical Research Council (MRC Clinical Research Training Fellowship G0902018, MRC Centenary Early Career Award, MRC Clinician Scientist Fellowship MR/M008517/1), Immune Tolerance Network and National Institutes of Health, and the Department of Health via the National Institute for Health Research (NIHR) comprehensive Biomedical Research Centre award to Guy’s & St Thomas’ NHS Foundation Trust in partnership with King’s College London and King’s College Hospital NHS Foundation Trust; has received lecture fees from Thermo Scientific, Nutricia and Infomed; has received travel support from European Academy of Allergy and Clinical Immunology (EAACI), British Society of Allergy and Clinical Immunology (BSACI), Academy of Medical Sciences, Portuguese Society of Allergy and Clinical Immunology (SPAIC), Spanish Society of Allergy and Clinical Immunology (SEAIC), and French Meeting of Molecular Allergology. H. A. Brough has received consultancy fees from MEAD Johnson Nutrition and Nutricia; has received research support from the Food Allergy Research and Education Charity grant, Immune Tolerance Network, and National Institutes of Health; has received lecture fees from MEDA Pharmaceuticals, Thermo Scientific, and Nutricia; receives royalties from Wiley Blackwell Ltd; has received travel support from British Society of Allergy and Clinical Immunology, MEDA Pharmaceuticals, Thermo Scientific, and Nutricia; and has received research support from Thermo Scientific, Stallergenes, and Meridien Foods.

Food allergy (FA) is an adverse reaction caused by an abnormal response of the immune system to food allergens. Food allergies are classified based on the involvement of IgE antibodies in their pathogenesis.^1^, ^2^ This review will focus on IgE-mediated FA. The immunologic mechanism underlying IgE-mediated allergy is type I hypersensitivity.^3^ During allergic sensitization, food allergens are presented to T cells, a Th2-skewed immune response commits B cells to IgE production and allergen-specific IgE binds to the high-affinity IgE receptors (FcεRI) on the surface of mast cells and basophils. In allergic individuals, on subsequent exposure to the allergenic food, multivalent allergens cross-link receptor-bound IgE leading to mast cell and basophil activation and the release of preformed mediators and *de novo* synthesis of leukotrienes and cytokines, which contribute to the symptoms that patients experience during allergic reactions.

Various *in vitro* assays reflect different aspects of the immunologic mechanisms of IgE-mediated FA. For instance, the amount of circulating allergen-specific IgE antibodies can be determined using immunoenzymatic assays, and basophil activation and T-cell proliferation in response to allergen can be assessed using flow cytometry (Figure 1). Some of these *in vitro* assays can be used to diagnose FA and/or defer or obviate the need for an oral food challenge (OFC). An OFC is the most accurate test to diagnose FA but requires expensive resources, highly trained personnel, and carries the risk of causing an acute allergic reaction. Therefore, in clinical practice, the diagnosis of FA is based on a combination of the clinical history and the results of allergy tests when possible. The clinical history, including the allergic reaction(s) to the culprit food and the dietary history, is the cornerstone of the diagnosis of FA; it guides the selection of allergens to be tested and the interpretation of allergy test results. In this review, we discuss the main *in vitro* tests for FA and how to make the most of these tests to decide whether an OFC is required to reach an accurate diagnosis of FA.

## *In Vitro* Tests for IgE-Mediated Food Allergy

### Specific IgE to allergen extracts

Specific IgE (sIgE) testing has been used to diagnose FA for many years. Automated systems permit the use of enzymatic immunoassays for a large number of samples in a standardized way; however, levels determined with different methodology may not be comparable.^4^ IgE is quantified using kilounits per liter (kU/L) based on the World Health Organization Reference Standard with 1 unit equaling 2.42 ng of IgE.^5^

Using the cutoff of 0.35 kU/L, sIgE testing has high sensitivity but poor specificity to diagnose FA. For example, in the case of peanut allergy (PA), sIgE to peanut has a sensitivity of 75% to 100% and a specificity of 17% to 63%.^6^, ^7^, ^8^, ^9^, ^10^, ^11^, ^12^, ^13^ Adopting 95% positive predictive value (PPV) cutoffs, the specificity of IgE testing increases. Following on with the example of PA, the cutoff of 15 kU/L^7^, ^14^ showed a specificity of 96.8% and a sensitivity of only 28.4% in a UK study.^7^ This indicates that the 95% PPV cutoffs can be useful to confirm the diagnosis of FA, especially if there is a recent history of an immediate-type allergic reaction. On the contrary, the cutoff of 0.35 kU/L can be useful to exclude the diagnosis of FA as it has a high negative predictive value (NPV). Levels of sIgE between positive and negative cutoffs without a clear clinical history do not allow us to confirm or exclude the diagnosis, falling in the so-called immunological gray area.^15^, ^16^ Positive and negative cutoffs can be helpful in guiding the clinical diagnosis of FA; however, they are not absolute and need to be interpreted in light of the clinical history, as patients can still be allergic or tolerant below and above 95% NPV and 95% PPV, respectively.^16^ PPV and NPV decision levels have been identified for sIgE to other foods (Table I).

Diagnostic cutoff values can vary widely in different studies. For instance, the 95% PPV cutoff to diagnose PA was 15 kU/L in US^14^ and UK^7^ studies, but was 24.1 kU/L, 34 kU/L, and 57 kU/L in studies performed in the Netherlands,^17^ Australia,^18^ and France,^19^ respectively. These differences can result from the patient population (eg, prevalence of FA, comorbidities) and/or from the research study where the cutoffs were determined (eg, inclusion criteria, reference standard against which the performance of sIgE was compared, criteria for referring for OFC and the OFC protocol).^20^ These factors need to be taken into account when comparing studies and when extrapolating cutoffs from published studies into daily clinical practice. When critically reviewing the literature for diagnostic decision levels for FA one should take into consideration the limitations of studies assessing the diagnostic utility of allergy tests (eg, small sample size, selected sample of participants, OFC not done in all participants, etc.).^21^ Validated diagnostic cutoffs are reliable when applied to a similar population to the population in which they were generated. PPVs are a function of the sensitivity and specificity of the test and the prevalence of the disease; therefore, they are only valid for patients who have the same pretest probability of disease as the population in which the PPV was established. For instance, in our clinic population in London, the cutoff of 15 kU/L for peanut sIgE had 95% PPV in 2 different studies performed approximately 10 years apart.^7^, ^16^ The consistency of these findings indicates that the identified cutoff can be reliably applied to our patient population in the clinic.

### Specific IgE to allergen components

Conventional IgE testing uses natural extracts containing a complex mixture of proteins. Allergen sIgE to component allergen tests for IgE binding to single allergens, allowing more precise profiling of the allergen-sIgE repertoire. The list of allergenic molecules available for testing is not complete; thus IgE assays using extracts are likely to be useful for some time. sIgE testing to components is available for single allergens and for multiple allergens in microarrays. Multiplex assays may introduce concerns where they reveal sensitization to molecules with potentially no clinical relevance as they are all tested independent of the patient's history. However, multiplex assays can be useful in identifying patterns of sensitization in complex polysensitized patients (eg, patients sensitized to pollen, plant foods, and latex with unclear clinical relevance that are sensitized to a panallergen) and in identifying the culprit allergen in patients with recurrent anaphylaxis.^22^, ^23^

The food that has received the most research into component allergens and their validation in terms of clinical relevance is peanut. The number of identified peanut allergens is extensive although not all of these are available for testing in clinical practice (Table II). The immunodominant peanut allergen in adults and children is Ara h 2 based on OFC, serial skin prick test (SPT) dilutions, and basophil degranulation assays.^24^, ^25^, ^26^, ^27^ Secondary sensitization to peanut occurs because of panallergens such as nonspecific lipid-transfer proteins (nsLTPs) (eg, Pru p 3 in peach giving rise to Ara h 9 sIgE), Bet v 1 homologs (eg, Bet v 1 in birch pollen giving rise to Ara h 8 sIgE), and profilins (eg, Phl p 12 in grass pollen or Bet v 2 in birch pollen giving rise to Ara h 5 sIgE). Similar to what has been reported for peanut sIgE, diagnostic cutoffs for Ara h 2 sIgE vary between studies (Table III).

Of the tree nuts, hazelnut has received the most extensive evaluation leading to the identification of seed storage proteins (eg, Cor a 9 and Cor a 14) as well as cross-reactive proteins (eg, Cor a 8 and Cor a 1) as allergens. sIgE to whole hazelnut has a poor predictive value for clinical reactivity due to cross-reactivity with birch pollen. Birch pollen-associated hazelnut allergy is the dominant phenotype, although Cor a 9 and 14 are the allergens more commonly associated with systemic reactions (Table III).^27^, ^28^, ^29^, ^30^ In Danish,^31^ German,^27^ and Belgian children^32^ Cor a 14 was superior to Cor a 9 in predicting challenge-proven hazelnut allergy; however, in Dutch children Cor a 9 was the best predictor.^28^ It was postulated that these differences were due to the age of children assessed with Cor a 9 specificity decreasing with age and Cor a 14 specificity increasing with age.^28^, ^33^ Other 2S albumins have been identified for walnut (Jug r 1),^34^, ^35^ cashew (Ana o 3),^36^ and Brazil nut (Ber e 1)^37^ (Table III).

Casein (Bos d 8), beta-lactoglobulin (Bos d 5), and alpha-lactoglobulin (Bos d 4) are the major allergens in cow's milk. Sensitivity to various cow's milk proteins is widely distributed; thus generally no single allergen is considered to be immunodominant.^38^ In some studies, Bos d 8 was the best predictor of challenge-proven cow's milk allergy (CMA).^39^, ^40^ In a Spanish study, the optimum cutoffs for Bos d 8 increased with age; using 2 kU/L (13-18 months), 4.2 kU/L (19-24 months), and 9 kU/L (24-36 months) gave a sensitivity of 95% and a specificity of 90%.^41^ This observation is important with regard to cutoffs for transient food allergies, such as cow's milk and egg, as one would expect that children who persist with CMA beyond 2 years would have higher casein levels than those who have already grown out of their CMA. In fact, IgE antibodies directed against sequential casein epitopes are a marker of persistent CMA.^42^ High casein-IgE antibodies are predictive of baked CMA as casein is more resistant to extensive heating.^43^ Clinical decision points for a positive challenge to baked milk have been reported (Table III).^44^

The main hen's egg allergens are ovomucoid (Gal d 1), ovalbumin (Gal d 2), conalbumin (Gal d 3), and lysozyme (Gal d 4).^45^ Ovomucoid is considered to be the immunodominant allergen based on OFCs to heated and ovomucoid-depleted egg^46^ and serial dilutions of ovomucoid SPT and ovomucoid sIgE in egg allergic children.^47^ Ovomucoid is stable against heat and digestion by proteinases^46^; this is why it has been evaluated in the prediction of tolerating extensively heated egg (Table IV). IgE antibodies to sequential epitopes of ovomucoid have been shown to predict persistent egg allergy beyond the age of 11 years.^48^

Component-resolved diagnosis (CRD) of wheat allergy has gained interest as wheat extract IgE testing has a poor predictive value. The major wheat allergens relevant for FA (rather than Baker's asthma) are glutens that can be subdivided into gliadins (subunits α, β, γ, and ω) and glutenins (high molecular and low molecular weight). The role of omega-5-gliadin (Tri a 19) in wheat-dependent exercise-induced anaphylaxis has been shown in several studies^49^, ^50^; however, results for this component in the prediction of IgE-mediated wheat allergy are conflicting. In a Japanese population, Tri a 19 has been shown to correctly predict challenge-proven IgE-mediated allergy to wheat,^51^, ^52^ and in a Swedish population, Tri a 19 correlated better with OFC-proven IgE-mediated wheat allergy than the extract-based *in vitro* test or other component allergens.^53^ However, the results for Tri a 19 have not been reproduced in American or German populations.^54^

Allergens predictive of systemic reactions to soya include the seed storage proteins Gly m 5, 6, and 8. Gly m 5 and 6 predicted systemic allergic reactions to soy (with both positive Gly m 5 and 6 giving an odds ratio of 12 for severe reactions) more than the Bet v 1 homolog Gly m 4.^55^ More recently, the 2S albumin Gly m 8 was found to be a better marker for systemic reactions to soy than Gly m 5 and 6 (or soy extract), but it still misclassified many patients.^56^, ^57^, ^58^ It is important to note that sole reactivity to the PR-10 protein Gly m 4 has been responsible for anaphylaxis after consumption of unprocessed soya.^59^

### Ratios: allergen-specific/total IgE and allergen-specific IgG4/IgE

To try to improve the diagnostic performance of food sIgE, the added value of total IgE and food-specific IgG4 has been tested in food-specific/total IgE ratios or food-specific IgG4/IgE ratios. Some studies showed an improvement in the prediction of OFC outcome with specific/total IgE ratios compared with sIgE alone,^60^ although other studies did not found it to be useful.^61^ The discrepancy in these findings could be due to the foods studied, as Gupta et al^60^ found the specific/total IgE ratio particularly useful for persistent food allergies (eg, peanut, tree nuts, shellfish, and seeds) and the study by Mehl et al^61^ focused on transient food allergies, namely, cow's milk, egg, and wheat allergies. Recently, in a multicenter study of children with suspected peanut or hazelnut allergies,^62^ calculating the Ara h 2/peanut sIgE or Ara h 2-specific/total IgE ratios did not improve the diagnostic performance of Ara h 2 sIgE. Peanut-specific/total IgE was also not better than Ara h 2 sIgE in diagnosing PA. Similar results were reported for the relative diagnostic performance of Cor a 14/hazelnut sIgE, Cor a 14-specific/total IgE, and Cor a 14 sIgE and hazelnut-specific/total IgE to diagnose hazelnut allergy.

Food-specific IgG4/IgE ratios have been determined in various studies, but their diagnostic utility has not been established. Sensitized-tolerant children tend to have higher allergen-specific IgG4/IgE ratios than allergic children. For instance, peanut-sensitized tolerant patients have a higher peanut-specific as well as Ara h 1-, Ara h 2-, and Ara h 3-specific IgG4/IgE ratios compared with peanut allergics.^63^ This increased IgG4 in relation to IgE was not due to higher peanut consumption as the majority of children had not knowingly eaten peanut before entering the study. A higher peanut-specific IgG4/IgE ratio has also been observed in peanut allergic patients treated with peanut oral immunotherapy^63^ and in high-risk infants who consumed peanut early in life and developed tolerance.^64^ Conversely, allergic patients tend to show a higher food sIgE/IgG4 ratio. For example, egg allergic patients who react to baked egg have higher ovalbumin and ovomucoid sIgE/IgG4 ratios than egg allergic patients who tolerate baked egg.^65^

### Basophil activation test

The basophil activation test (BAT) is a functional assay that uses live basophils in whole blood to detect the ability of IgE to mediate activation of basophils after stimulation with allergen. It goes beyond the detection of IgE binding to allergen to test IgE function, which depends not only on the allergen-sIgE levels but also on IgE epitope specificity, affinity, and clonality.^66^ The basophils of allergic patients typically show a dose-dependent expression of activation markers, such as CD63 or CD203c, whereas the basophils of sensitized-tolerant patients do not express or have a much lower expression of activation markers after stimulation with allergen. In a PA study, basophils of peanut allergic patients showed higher basophil activation to peanut compared with peanut-sensitized-tolerant even in the subgroup where allergic and tolerant children had comparable levels of peanut sIgE.^16^ The difference in upregulation of basophil activation markers in response to allergen between allergic and nonallergic patients forms the basis of the use of the BAT to diagnose FA.

The main added value of the BAT in the diagnosis of FA compared with tests routinely used in clinical practice, such as SPT and sIgE to allergen extracts, is its enhanced specificity with often conserved sensitivity. For instance, the BAT to peanut showed 98% sensitivity and 96% specificity to diagnose PA, with the specificity reaching 100% in a subsequent validation. The specificity of the BAT ranged between 77% and 100% in other studies (Table IV).^11^, ^67^, ^68^, ^69^, ^70^, ^71^, ^72^, ^73^ The BAT with single allergen components can potentially improve its diagnostic accuracy, but further research studies are needed.^72^, ^74^, ^75^ The BAT has been shown to be potentially useful in identifying the culprit allergen in cases of pollen-food allergy syndrome (PFAS),^71^, ^76^, ^77^ allergy to red meat,^78^ or food-dependent exercise-induced anaphylaxis.^79^ As for other diagnostic tests, cutoffs determined for the BAT can vary with the patient population, the design of the study, and the methodology adopted for the BAT procedure and data analyses.^20^

The BAT requires fresh blood and uses flow cytometry for which appropriate equipment and trained personnel are needed. It is anticipated that the BAT is reserved for selected cases where the results of routinely used tests do not allow a precise diagnosis. Indeed, in the previously mentioned study,^16^ the BAT sustained its good performance in a subgroup of patients with equivocal test results for SPT, peanut sIgE, and Ara h 2 sIgE with 92% accuracy compared with its 97% accuracy in the study population overall. Used as a second step in the diagnostic workup, the BAT was performed in patients who would have otherwise been referred for an OFC after standard allergy testing. A positive BAT confirmed the diagnosis of FA and dispensed with an OFC, whereas patients with a negative BAT or nonresponder basophils (ie, basophils that solely responded to non–IgE-mediated and not to IgE-mediated stimulants) needed to be referred for the OFC. This stepwise approach ensured a 67% reduction in the need for the OFC.^16^

As any other diagnostic test, the BAT cannot be used in isolation to diagnose FA. The results of the BAT need to be considered in light of the clinical history. In addition to patients with a negative BAT or nonresponder basophils, patients with BAT results that are discordant with the clinical history require an OFC to confirm or refute the diagnosis of FA.

### IgE to allergen peptides

IgE specificity can be refined further by determining the allergen epitopes to which IgE binds. This has been evaluated using short linear allergen peptides of 15 to 20 amino acids bound to a solid phase (eg, microarray or spot membrane) using immunofluorescence. Beyer et al^80^ identified 5 immunodominant epitopes in selected peanut allergen peptides in 2003. Years later, a microarray containing peptides of the major peanut allergens, Ara h 1, Ara h 2, and Ara h 3, identified epitopes bound more by the IgE of peanut allergic patients than by the IgE of peanut sensitized-tolerant patients; this allowed the development of a machine-learning method that markedly enhanced the diagnostic utility of the microarray.^81^

Similar methods have tested the utility of IgE to allergen peptides in diagnosing and in predicting the resolution of other food allergies.^82^, ^83^, ^84^, ^85^, ^86^ In a CMA study,^82^ IgE binding was more diverse and had higher affinity for cow's milk allergen peptides in milk allergic patients reacting to baked milk compared with patients who reacted to unheated milk but tolerated baked milk, suggesting that the peptide microarray could be useful in identifying different phenotypes of CMA.

### T-cell assays

T-cell responses are central to the development of oral tolerance in nonallergic individuals and to the development of the allergic immune response in allergic individuals. Peanut allergic individuals have been shown to have greater proliferation of their T cells when their PBMCs were stimulated with whole peanut or individual major peanut allergens.^87^, ^88^ Peanut allergic patients also showed a typical Th2-skewed response to peanut allergen with higher levels of IL-4, IL-5, and IL-13, whereas nonallergic controls showed a Th1-type response characterized by IFN-gamma production.^87^ Interestingly, peanut allergic and peanut-sensitized-tolerant individuals showed higher T-cell proliferation compared with nonsensitized controls; however, only allergic patients showed a Th2-skewed response to peanut allergens.^88^ These findings suggest that the absence of clinical reactivity in sensitized individuals is an active ongoing process, whereas in nonsensitized individuals, it is a passive process, probably due to anergy or clonal deletion.

Food allergic patients may also have impaired regulatory T-cell function in response to specific food allergens. Dang et al^89^ recently showed that egg and/or peanut allergic infants had a reduction in the number of T regulatory cells and a lower ratio of activated regulatory/effector T cells *in vitro* after *in vivo* allergen exposure during the OFC. This is consistent with studies in mouse models.^90^

## Clinical Reasoning to Diagnose Food Allergy

The tests available for routine use in the clinic can vary, with some practices using mainly SPT, others mainly sIgE, and others both. sIgE to allergen components is used in most specialist centers and the BAT is becoming increasingly used clinically. The other tests described in the previous section are reserved for use in the research setting, namely, peptide microarrays and T-cell assays.

### Interpretation of allergy test results

The ultimate goal of the allergy test result is to determine the probability of clinical allergy; this is then used to decide whether an OFC is warranted.^91^ The probability of clinical allergy depends first and foremost on the clinical history (Table V) and secondarily on the allergy test result (Tables I, III, and IV). For example, if a patient consumes age-appropriate amounts of the food regularly without developing any symptoms, the probability of having FA is negligible regardless of the allergy test results; such patients should in fact not be tested as a false-positive result could be confusing for the patient and lead to unnecessary food avoidance. The clinical history provides information that enable the clinician to establish a pretest probability of FA that will be taken into account to determine the probability of clinical allergy for a given allergy test result, that is, the post-test probability.^91^ This reasoning is best described using nomograms that use likelihood ratios to calculate the post-test probability based on a given pretest probability. Likelihood ratios have the advantage of not depending on the prevalence of the disease in the population, as opposed to PPV, and can be calculated from the sensitivity and specificity of the test.^91^, ^92^, ^93^ Different factors can modulate pretest probabilities and likelihood ratios, for instance, the previous allergic reaction(s), the dietary history, age, ethnicity, concomitant atopic diseases, geographical location, and the clinical setting. This is best studied for sIgE testing.

The clinical relevance of a given allergen sIgE result can vary depending on the age of the patient, with lower levels of sIgE having increased clinical relevance in younger patients.^94^ Ninety-five percentage PPV cutoffs have been established for children <2 years at lower levels of food sIgE compared with cutoffs for older children.^14^, ^95^

Diagnostic decision levels may be affected by the patients' ethnicity. Black race is associated with a higher prevalence of sensitization to foods^96^ and a higher level of total IgE compared with Caucasians^97^ despite lower prevalence of FA.^96^ This discrepancy suggests that patients of black ethnicity may have more clinically irrelevant IgE and therefore higher diagnostic cutoffs. Indeed, the 95% cutoffs defined in the United Kingdom^7^ for peanut sIgE and Ara h 2 sIgE provided lower PPVs in South African peanut-sensitized patients^98^; the optimal cutoffs to diagnose PA in this population were ≥15 kU/L for peanut sIgE and ≥8 kU/L for Ara h 2 sIgE, which had 80% and 93% PPV, respectively.

Concomitant atopic diseases can also modulate the clinical relevance of a given allergy test result. Patients with atopic eczema tend to have a polyclonal IgE response to allergens that often lacks clinical expression. This underscores the importance of a judicious selection of allergens to be tested. Grabenhenrich et al^62^ showed that for a given component-sIgE level, a high total IgE (>500 kU/L) significantly reduced the probability of clinical peanut or hazelnut allergy, respectively, particularly at low levels of Ara h 2 sIgE or Cor a 14 sIgE. In patients with birch or grass pollen allergy, high levels of sIgE to plant foods, such as peanut or hazelnut, may have a low probability of a systemic allergic reaction. These are the cases where determining sIgE to individual allergens that are involved in cross-reactivity (eg, Ara h 8 and Cor a 1) can be helpful in distinguishing real FA from sensitization secondary to pollen allergy, which can cause PFAS but usually not systemic allergic reactions.

Geographical location is another factor that may influence the clinical relevance of a given sIgE level. A study by Vereda et al^99^ illustrates this nicely for PA. In Northern and Central Europe, sensitization to birch pollen leads to high prevalence of sensitization to Ara h 8, the Bet v 1-homolog, which typically causes oral allergic symptoms. In Spain, exposure to birch pollen and sensitization to Ara h 8 are rare and peanut allergic patients are often sensitized to Ara h 9 (nsLTP), probably as a consequence of primary sensitization to peach LTP. In the United States and in the United Kingdom, the most common pattern of sensitization in peanut allergic patients is the combination of IgE to Ara h 1, Ara h 2, and Ara h 3, although other patterns may be found in individual patients.^63^

Finally, the clinical setting influences the predictive value of sIgE levels, with increasing likelihood of clinical allergy going from the general population to secondary care and then to specialist centers. In studies performed in the general population, the prevalence of sensitization to foods such as cow's milk and egg was much lower than in a population recruited from specialist centers. For example, approximately 8% and 3%-4% of children in National Health and Nutrition Examination Survey 2005-2006^100^ and 78% and 89% of children in Consortium of Food Allergy Research (COFAR)^101^ were sensitized to cow's milk and egg, respectively, although this is probably an extreme example as a positive SPT to cow's milk and/or egg was one of the inclusion criteria in COFAR, and therefore it is a highly selected population.

### Factors influencing the decision of performing an oral food challenge

The main reason to perform an OFC is to identify the food that caused the allergic reaction for the initial diagnosis and for monitoring resolution of FA. Other reasons for an OFC include assessing the status of tolerance to cross-reactive foods (eg, tree nuts in PA or peanut in egg allergy) and expanding the diet in foods not yet introduced but with positive allergy tests. This occurs more frequently because of the increased use of anticipatory testing and has important resource implications.^102^

Many factors affect the decision as to whether to perform an OFC (Table VI). The most important considerations are provided by the clinical history; the previous reaction history and recent exposure to the food in question may avert the need (already consuming) or lead to deferment (recent reaction, poorly controlled asthma) of an OFC. SPT and sIgE testing will also affect this decision; in a patient being assessed for the initial diagnosis of FA, a recent convincing history of an allergic reaction to an identified food, concomitant SPT ≥ 3 mm and/or sIgE to the whole allergen ≥0.35 kU/L may be sufficient to confirm the diagnosis without the need for further testing or an OFC. In cases where the history and SPT/sIgE testing do not provide a clear answer, further testing with sIgE to components or the BAT may be warranted, before deciding to perform an OFC.

When monitoring a patient for resolution of FA, it is generally recommended that children with a 50% chance of experiencing a negative challenge be good candidates for an OFC.^103^ A predictive 50% negative cutoff of 2 kU/L has been identified for the resolution of egg and cow's milk allergies.^104^ The rate of decline of IgE to cow's milk and egg has also been shown to predict resolution; a 50% decrease in respective sIgE over 12 months is associated with a 52% probability of tolerance to egg and 31% probability of tolerance to cow's milk.^105^ Baseline sIgE and SPT wheal size and severity of eczema also affect the rate of resolution and this has been incorporated into a practical computerized algorithm by Wood et al^106^ for CMA. In the case of peanut, sIgE ≤2 kU/L and ≤5 kU/L have been shown to give a 50% prediction of a negative peanut challenge in children with and without a history of peanut reaction, respectively.^107^ A systematic review by Peters et al^108^ in 2013 provides further details on sIgE and SPT cutoffs to predict the resolution of cow's milk, egg, and peanut allergies.

When considering performing an OFC it is vital that the patient or parents of the child undergoing the OFC understand the rationale for this and the importance of introducing the food into the diet after a negative challenge. Several studies have shown that 18% to 32% of patients do not introduce the food after passing an OFC.^109^, ^110^, ^111^ This is of concern as the recurrence of FA (particularly peanut) has been shown to occur if the food continues to be avoided after the OFC or is consumed in very small quantities.^112^, ^113^, ^114^ This would suggest that the immune system needs ongoing exposure to maintain tolerance; however, this conflicts with the fact that children develop tolerance whilst avoiding a food. Nonetheless, if the food is not important to the patients and they are not planning to introduce it, then it may be better not to proceed with the OFC. Dietetic advice to prepare recipes that the child will accept and suggestions for foods for mixing can avert failed OFCs; dietitians can also advise on ways to introduce the food. Another important consideration is to avert failed OFCs due to the patient or family not being prepared for the OFC due to uncontrolled asthma, continued antihistamine use, and inability or refusal to complete the OFC. Clear verbal and written information before the OFC is therefore essential.^66^

### Severity

Identifying patients at high risk of a severe reaction to foods is important for the management of patients diagnosed with FA. Previous studies have shown contradictory results about the utility of food-specific IgE levels in assessing severity of FA.^8^, ^115^, ^116^ sIgE to certain allergen components, such as Ara h 2 in peanut, has been associated with more severe reactions than sIgE to whole peanut or other single allergens, which is corroborated by *in vitro* studies of basophil activation and mediator release assays where Ara h 2 and Ara h 6 have been shown to be the most potent elicitors of effector cell response.^25^ On the contrary, sIgE to Ara h 8 is associated with PFAS. Higher reactivity on the BAT to food allergens has been shown to be associated with greater severity of allergic reactions during an OFC.^117^, ^118^, ^119^ In a peptide microarray, a broader IgE epitope diversity is associated with more severe reactions and with a greater degree of basophil activation and degranulation after allergen stimulation.^120^, ^121^

The above data need to be applied with caution to the assessment of individual patients. For example, patients with raised Ara h 2 sIgE do not necessarily have severe PA and can actually pass a peanut OFC^122^; 10% of patients with PFAS can have systemic reactions and 1% to 2% experience anaphylaxis.^123^, ^124^ The risk assessment of allergic patients depends on factors other than mere individual players of IgE-mediated food-induced allergic reactions (such as single allergens or epitopes, IgE, or basophils) and requires a holistic clinical evaluation of the patient.

## Conclusions

*In vitro* allergy tests are useful in diagnosing IgE-mediated FA and support the decision of whether an OFC is necessary to reach an accurate diagnosis. Validated cutoffs are reliable when applied to a similar patient population to the one where they were developed. Patient-specific factors can modulate the probability of clinical allergy of a given sIgE result. IgE to allergen components can provide more precise information about IgE specificity. The BAT assesses the function of IgE in its ability to mediate allergen-induced effector cell activation. Further research is needed to improve our understanding about how the information of various tests can be combined for optimal diagnostic accuracy to reduce the need to perform OFCs to a minimum.

## Figures and Tables

**Figure 1:**
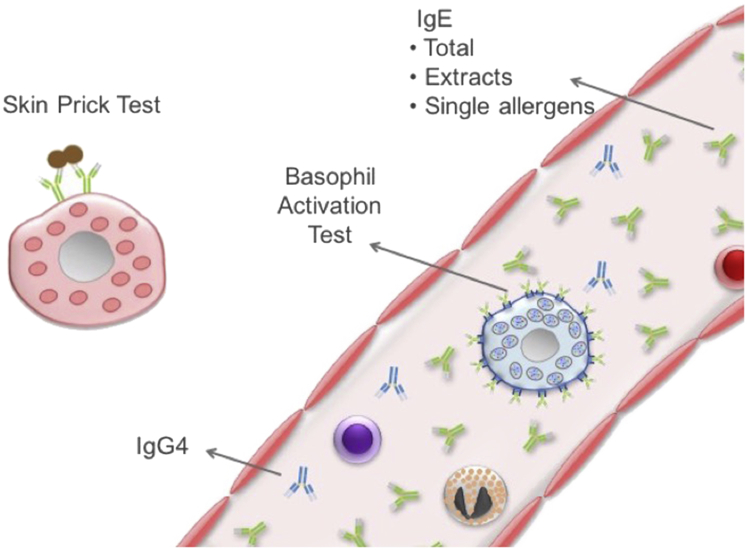
Tests used to diagnose IgE-mediated food allergy reflect different aspects of the underlying mechanism of this immune-mediated disorder: the skin prick test measures the response of skin mast cells to allergen, the basophil activation test measures the response of circulating basophils to allergen, and IgE tests measure the concentration of circulating IgE, either total IgE or sIgE to allergen extracts or to individual allergen components. Total IgE and allergen-specific IgG4 can be used to calculate ratios with allergen sIgE.

**Table I:** Examples of diagnostic cutoffs with 95% PPV and 50% NPV for specific IgE to food allergen extracts^14^, ^107^, ^125^

Approximate predictive value	Cow's milk	Egg	Peanut	Fish
95% PPV	32 kU/L	7 kU/L	15 kU/L	20 kU/L
50% NPV	2 kU/L	2 kU/L	2 kU/L∗	–
			5 kU/L∗	

*NPV*, Negative predictive value; *PPV*, positive predictive value.

∗ The 50% NPV cutoff is different depending on the previous history of reaction: 2 kU/L if the patient reports a reaction and 5 kU/L if the patient has never had an allergic reaction to peanut in the past.

**Table II:** Peanut allergens described to date^126^

Allergen	Biochemical name
**Ara h 1**	**Cupin (Vicillin-type, 7S globulin)**
**Ara h 2**	**Conglutin (2S albumin)**
**Ara h 3**	**Cupin (Legumin-type, 11S globulin, Glycinin)**
Ara h 4	Considered an isoform of Ara h 3 and renamed to Ara h 3.02
Ara h 5	Profilin
Ara h 6	Conglutin (2S albumin)
Ara h 7	Conglutin (2S albumin)
**Ara h 8**	**Pathogenesis-related protein 10 (PR-10, Bet v 1 homolog)**
**Ara h 9**	**Nonspecific lipid-transfer protein type 1**
Ara h 10	Oleosin
Ara h 11	Oleosin
Ara h 12	Defensin
Ara h 13	Defensin
Ara h 14	Oleosin
Ara h 15	Oleosin
Ara h 16	Nonspecific lipid-transfer protein type 2
Ara h 17	Nonspecific lipid-transfer protein type 1

Allergens in bold are commercially available for clinical use.

**Table III:** Allergen components associated with clinical allergy and examples of cutoffs for specific IgE testing to main allergen components

Foods	Components associated with clinical allergy	Cutoffs for specific IgE to main components
Peanut	Ara h 1	Ara h 2 sIgE: 0.35 to 42.2 kU/L had 90%-95% PPV^16^, ^24^, ^27^
	Ara h 2	
	Ara h 3	
	Ara h 9 (in Southern Europe)	
Hazelnut	Cor a 9	Cor a 9 sIgE: 1 kU/L had 83% accuracy^28^
	Cor a 14	Cor a 14 sIgE: 0.72 to 47.8 kU/L had 87%-90% accuracy^27^, ^31^
	Cor a 8 (in Southern Europe)	
Cashew, Pistachio	Ana o 3	Ana o 3 sIgE: 0.16 kU/L had 97.1% accuracy for cashew and/or pistachio nut allergy^127^
Brazil nut	Ber e 1	Ber e 1 sIgE: 0.25 kU/L had 94% PPV^128^
Walnut	Jug r 1	Jug r 1 sIgE: 0.1 kU/L had 91% PPV^129^
	Jug r 3	
Soya	Gly m 5	Gly m 8 sIgE: 1 kU/L had 89% PPV^56^
	Gly m 6	Gly m 8 sIgE: 0.1 kU/L had 83% NPV^56^
	Gly m 8	
Wheat	Tri a 19 (IgE-mediated wheat allergy and WDEIA)	Tri a 19 sIgE: 0.04 AU had 100% PPV and 88% NPV for IgE-mediated wheat allergy^51^, ^52^
	Tri a 14 (nsLTP involved in Baker's asthma)	
Cow's milk	Casein (for baked milk allergy and persistent cow's milk allergy)	Casein sIgE: 10 kU/L had 95% PPV for a positive OFC to baked milk^44^
		Casein sIgE: 5 kU/L had 50% PPV for a positive OFC to baked milk^44^
Egg	Ovomucoid (for cooked or baked egg allergy and persistent egg allergy)	Ovomucoid sIgE: 3.74-26.6 kU/L had 95% PPV for cooked egg allergy^130^, ^131^
		Ovomucoid sIgE: 50 kU/L had 90% PPV and Ovomucoid sIgE: 0.35 kU/L had 90% NPV for a positive OFC to baked egg^132^

*nsLTP*, Nonspecific lipid-transfer protein; *OFC*, oral food challenge; *WDEIA*, wheat-dependent exercise-induced anaphylaxis.

**Table IV:** Basophil activation test to food extracts or to component allergens in the diagnosis of food allergy

Food extract or allergen component	Cutoffs	Diagnostic performance					
		S	Sp	PPV	NPV	LR+∗	LR−∗
Cow's milk	SI CD203c ≥1.9^67^	89%	83%	86%	86%	5.24	0.13
	>6% CD63+^117^ to diagnose resolution of CMA	91%	90%	81%	96%	9.10	0.10
Casein	SI CD203c ≥1.3^67^	67%	71%	74%	63%	2.31	0.46
Egg white	SI CD203c ≥2.4^67^ to diagnose baked egg allergy	74%	62%	85%	44%	1.95	0.42
	SI CD203c ≥1.7^67^ to diagnose raw egg allergy	77%	63%	92%	33%	2.08	0.37
Ovalbumin	≥5% CD63+ or SI CD203c ≥1.6 to diagnose egg allergy	77% for CD63	100% for CD63			Inf†	0.23 for CD63
		63% for CD203c	96% for CD203c			15.75 for CD203c	0.39 for CD203c
Ovomucoid	SI CD203c ≥1.7^67^ to diagnose baked egg allergy	80%	73%	90%	53%	2.96	0.27
	SI CD203c ≥1.6^67^ to diagnose raw egg allergy	83%	83%	97%	42%	4.88	0.20
Wheat	>11.1% CD203c+ to diagnose wheat allergy^68^	86%	58%	77%	71%	2.05	0.24
Omega-5 gliadin	nTri a 19: >14.4% CD203c+ to diagnose wheat allergy^68^	86%	58%	77%	71%	2.05	0.24
	rTri a 19: >7.9% CD203c+ to diagnose wheat allergy^68^	83%	63%	81%	67%	2.24	0.27
Peanut	≥4.78% CD63+^16^	98%	96%	95%	98%	24.50	0.02
Ara h 1	ND	BAT to Ara h 1 was higher in peanut allergic patients compared with controls from Southern Spain^74^					
Ara h 2	ND	92%	77%			4.00	0.10
Ara h 3	ND	There was no difference in BAT to Ara h 3 between peanut allergic and control subjects from Southern Spain^74^					
Ara h 6	ND	There was no difference in BAT to Ara h 6 between peanut allergic and control subjects from Southern Spain^74^					
Ara h 8	ND	There was no difference between CD-sens to Ara h 8 between patients with PFAS to peanut and patients with sIgE to Ara h 8 and no reaction during OFC to roasted peanuts^76^					
Ara h 9	ND	BAT to Ara h 9 was higher in peanut allergic patients compared with controls from Southern Spain^74^					
Hazelnut	CD-sens >1.7^69^ to diagnose hazelnut allergy	100%	97%			33.33	0.00
	≥6.7% CD63+^70^ to diagnose PFAS to hazelnut	85%	80%			4.25	0.19
Peach	>20% CD63+ and SI CD63 >2^75^	87%	69%			2.81	0.19
Pru p 3	>20% CD63+ and SI CD63 >2^75^	77%	97%			25.67	0.24
Apple	≥17% CD63+^71^ to diagnose PFAS to apple	88%	75%			3.52	0.16
Carrot	≥8.9% CD63+^70^ to diagnose PFAS to carrot	85%	85%			5.67	0.18
Celery	≥6.3% CD63+^70^ to diagnose PFAS to celery	85%	80%			4.25	0.19

*BAT*, Basophil activation test; *CMA*, cow's milk allergy; *Inf*, infinity; *LR+*, positive likelihood ratio; *LR−*, negative likelihood ratio; *ND*, not determined; *NPV*, negative predictive value; *OFC*, oral food challenge; *PFAS*, pollen-food syndrome; *PPV*, positive predictive value; *S*, sensitivity; *SI*, stimulation index; *Sp*, specificity.

∗ Likelihood ratios were calculated from sensitivity and specificity using the formulas LR+ = sensitivity/(1 − specificity) and LR− = (1 − sensitivity)/specificity.

† Infinity, the denominator is zero.

**Table V:** Factors modulating the interpretation of allergy test results

Factors identified in the clinical history	Effect on the probability of clinical allergy for a given specific IgE level	
Reported immediate allergic reaction to the specific food		A history of reacting to the tested food supports the clinical relevance of detected IgE.
(Younger) Age		Lower levels of allergen-specific IgE have increased clinical relevance in young children.
(Black) Ethnicity		Black race is associated with higher levels of allergen-specific IgE with decreased clinical relevance.
Atopic eczema		Polyclonal IgE response can be non-allergen-specific and thus decrease clinical relevance of a given specific IgE level.
Concomitant inhalant allergies		Pollen sensitization can cause false-positive results of specific IgE to plant food extracts.
Atopic population		Positive predictive value of a given specific IgE level increases with the increase in the prevalence of the disease in the population.
Geographical location	Variable	Clinical relevance of IgE to extracts and patterns of sensitization to allergen components can vary with inhalant allergen exposure typical of certain geographical locations.

These factors affect the pretest probability and therefore influence the resulting post-test probability.

**Table VI:** Factors influencing the decision to perform an oral food challenge (OFC)

Factors	Effect on the decision to perform an OFC	
History of an allergic reaction		A previous history of a reaction to the specific food increases the chance of reacting during the OFC.
Recent exposure to the food		A recent allergic reaction or the consumption of age-appropriate amount of the food precludes the OFC.
(Low) specific IgE levels		Current low level of food-specific IgE and >50% decline within the last year indicate lower likelihood of a positive OFC.
Importance of the food		The importance of the food to the child's diet and social life and her or his willingness to eat the food regularly in the case of a negative challenge favor performing an OFC.
Resources available		The resources available may limit the number of OFCs offered to patients.
Patient preferences	Variable	Patient may wish to undergo an OFC or not and her or his preferences need to be taken into account.

The decision to perform an OFC is made when the probability of a systemic reaction is sufficient for there to be concern and low enough that the OFC is likely to be passed. The arrows indicate the effect on the decision to perform an OFC: the arrow pointing up means weighing pro and the arrow pointing down means weighing con performing an OFC.
