# Correction

**DOI:** 10.1016/j.jaip.2017.03.008

**Published:** 2017

With regard to the article in the March/April 2017 issue entitled “Making the Most of *In Vitro* Tests to Diagnose Food Allergy” (J Allergy Clin Immunol: In Pract 2017;5:237-48), reference 48 was incorrectly listed. It should have read “Järvinen KM, Beyer K, Vila L, Bardina L, Mishoe M, Sampson HA. Specificity of IgE antibodies to sequential epitopes of hen's egg ovomucoid as a marker for persistence of egg allergy. Allergy 2007;62:758-65.” The authors regret this error.

